# Renal insufficiency plays a crucial association factor in severe knee osteoarthritis-induced pain in patients with total knee replacement: A retrospective study

**DOI:** 10.1097/MD.0000000000019125

**Published:** 2020-02-07

**Authors:** Chung-Ching Chio, Man-Kit Siu, Yu-Ting Tai, Tyng-Guey Chen, Wei-Pin Ho, Jui-Tai Chen, Hsiao-Chien Tsai, Ruei-Ming Chen

**Affiliations:** a Department of Neurosurgery, Chi Mei Medical Center, Tainan; b Cell Physiology and Molecular Image Research Center, Wan Fang Hospital; c Graduate Institute of Medical Sciences, College of Medicine, Taipei Medical University, Taipei, Taiwan; d Department of Anesthesiology, Centro Hospitalar Conde de Sáo Januário, Macau Special Administrative Region, China; e Department of Anesthesiology, Wan-Fang Hospital; f Department of Anesthesiology, Shuang Ho Hospital; g Anesthesiology and Health Policy Research Center, Taipei Medical University Hospital, Taipei Medical University; h TMU Research Center of Cancer Translational Medicine, Taipei, Taiwan.

**Keywords:** estimated glomerular filtration rate, osteoarthritis, renal insufficiency, serum creatinine, severe knee pain

## Abstract

Pain, the main symptom of osteoarthritis (OA), can lead to functional disability in patients with knee OA. Understanding the association factors related to knee pain is important since preventing OA-induced disabilities can be achieved by modifying these pain-associated issues. Therefore, this study was aimed to investigate the association factors for OA-induced knee pain in Taiwanese patients who received total knee replacements (TKR).

In this retrospective study, 357 subjects who had undergone TKR at the Taipei Municipal Wan-Fang Hospital were recruited. The distribution of pain severity among patients with knee OA was evaluated. Demographic data and clinical parameters were analyzed to determine relationships between these variables and the severity of knee OA pain.

Of the 357 patients studied, 54% and 33% had moderate and severe knee pain, respectively. Furthermore, a multivariate logistic regression analysis revealed that serum creatinine (>1.5 mg/dL) and an estimated glomerular filtration rate (eGFR) (<60 mL/min/1.73 m^2^) were significantly associated with severe knee pain in OA patients. A significant correlation between severe knee pain and serum creatinine or eGFR was demonstrated by Pearson correlations.

Taken together, the renal insufficiency defined by an elevated serum creatinine or a low eGFR in OA patients who required TKR was associated with severe knee pain. These variables must be considered while treating knee OA pain, especially in those patients with severe pain.

## 1 Introduction

Knee osteoarthritis (OA) is considered a degenerative joint disease involving progressive cartilage degeneration, subchondral bone changes, osteophyte formation, and synovitis.^[1,2]^ OA more commonly presents in women than men, and its prevalence is also related to aging.^[3]^ In Asian populations, such as southern China, the prevalence of knee OA is about 13% and even higher in rural areas.^[4–6]^ In patients aged over 65 years in Taiwan, the prevalence of knee OA was 9.6% in 2005, and may rise to 14% in 2020.^[7]^ In the global health, the prevalence of OA is increasing due to population aging and an increase in related factors such as obesity.^[8]^ An estimated 10% to 15% of all adults aged over 60 have some degree of OA. Thus, OA becomes a challenged health issue around the world.

Symptoms of knee OA are joint pain, stiffness, muscle weakness, and a limited range of motion.^[2,9]^ Furthermore, severe knee pain makes patient disabled, limiting their daily functions, and impairing their quality of life.^[10,11]^ Pain management of OA includes analgesic medication of nonsteroidal anti-inflammatory drugs (NSAIDs), intra-articular steroids, acupuncture, and physical therapy.^[12]^ A total knee replacement (TKR) is a final procedure to treat patient with symptomatic knee OA or severe knee pain after failing pain relief treatments.^[2,13]^ Nonetheless, the severity of knee pain and functional disability are the strongest factors for patients to receive a TKR.

Knee pain is the main compliant of knee OA patients, and it is a leading cause to make functional disability.^[14]^ A major symptom is pain that is triggered by peripheral and central changes within the pain pathways.^[13,15]^ Reported risk factors for knee pain in previous studies included a female gender, age, obesity, radiographic OA, previous knee injuries, and knee-straining work.^[16,17]^ Understanding the risk factors related to the OA knee pain and its severity is necessary because prevention of the pain-induced disabilities can be achieved by modifying those associated factors. As a result, this retrospective study was aimed to investigate the association factors for knee OA pain in those patients who have received TKR surgery.

## 2 Material and methods

### 2.1 Study patients

In this retrospective study, we collected information on clinical characteristics of patients at the Wan Fang Medical Center, Taipei Medical University, Taipei, Taiwan, from 2006 to 2012. This study was approved by the joint-institutional review board of the Taipei Medical University. We included 357 TKR patients aged over 45 years old with knee pain, and with radiographic evidence of an OA knee, or an orthopedist-diagnosed OA knee according to American College of Rheumatology criteria.^[18]^ We excluded those who had a knee trauma history, rheumatoid arthritis, septic arthritis or revision TKR on the ipsilateral side of the OA knee. From patients’ medical records, we gathered data of demographic information, knee pain severity, and clinical variables (such as preoperative hemoglobin, and serum creatinine levels).

### 2.2 Demographic data

We included patients’ age, gender, body height, body weight, body mass index (BMI) and also information on the presence or absence of cardiovascular disease, diabetes mellitus, and hypertension according to physicians’ diagnoses. The smoking status was also identified as a former/current smoker, or nonsmoker. Data of preoperative laboratory blood chemistry of serum creatinine and serum hemoglobin were obtained. Anemia was defined that serum hemoglobin level of <13 g/dL in men, and <12 g/dL in women, according to the criteria of the World Health Organization (WHO).^[19]^ To define renal insufficiency, we used cut-off values of the estimated glomerular filtration rate (eGFR) <60 mL/min per 1.73 m^2^ (calculated by the equation of the Modification of Diet in Renal Disease), and serum creatinine exceeding 1.5 mg/dL.

### 2.3 Assessment of knee pain

Knee pain was assessed by reviewing a patient's medical records. When a patient was admitted, the worst knee pain on the OA knee within 1 month before admission was rated by the patient, using a 10-point numeric rating scale (NRS), from no pain (0) to the worst pain (10). Based on NRS scores, patients’ pain severity was classified into 3 groups: (mild pain: (0–3), moderate pain: (4–6), and severe pain: NRS (7–10), the cut-off value between each severity level was based on a previously published report.^[20]^

### 2.4 Knee imaging examination

A knee radiographic imaging examination, using an anterior-posterior view in a weight-bearing position, was obtained before the operation in each patient. The severity of the radiographic knee OA was defined by the Kellgren–Lawerence (K/L) grading scale: grade 0 (normal); grade 1 (doubtful osteophytes); grade 2 (definite osteophytes and possible narrowing of the joint space); grade 3 (moderate multiple osteophytes, definite narrowing of the joint space, some sclerosis, and possible deformity of the bone contour); grade 4 (large osteophytes, marked narrowing of the joint space, severe sclerosis, and definite deformity of the bone contour).^[21]^ Greater than grade 1 was defined as radiographic knee OA. One well-trained rater who was blinded to this study's subjects, read all of the knee radiographic images, and reported the K/L grading scales.

### 2.5 Statistical analysis

We performed data analysis with the Statistical Package for Social Science vers.17.0 (, Chicago, IL). Demographic information (sex gender, age, body height, body weight, and BMI), bilateral OA knee history, past history of heart disease, diabetes mellitus, hypertension, imaging evidence of OA knee severity, hemoglobin and creatinine data were evaluated by a 1-way analysis of variance to determine differences between individual OA knee pain group. We used a multivariate binary logistic regression to evaluate the adjusted odds ratio (OR) of factors associated with severe knee pain, compared to mild and moderate knee pain. A stepwise regression was performed when a logistic regression was used. Reference categories included male, aged <65 years, BMI <25 kg/m^2^, no bilateral OA knee history, nonsmoker, OA severity grade II and without heart disease, diabetes mellitus, hypertension, perioperative anemia, or renal insufficiency. A significant correlation was determined by Pearson correlation test. Differences were considered significant if *P*-values were <.05.

## 3 Results

### 3.1 Baseline characteristics of studied subjects

This study included 357 knee OA participants with knee pain who had undergone TKR surgery at the Wan Fang Medical Center, Taipei Medical University, from 2006 to 2012. Table 1 gives demographic data of subjects. The mean age of knee OA patients was 71.5 ± 8.0 years. Seventy-nine percent of subjects were females. The mean BMI was 28.16 ± 4.47 kg/m^2^. On radiographic assessment, according the K/L grading, 34 (9.5%) patients were defined as grade 2, 171(47.9%) patients as grade 3, and 152 (42.6%) patients as grade 4. Patients planning to receive TKR were mostly categorized into moderate to severe radiographic OA grades.

**Table 1:**
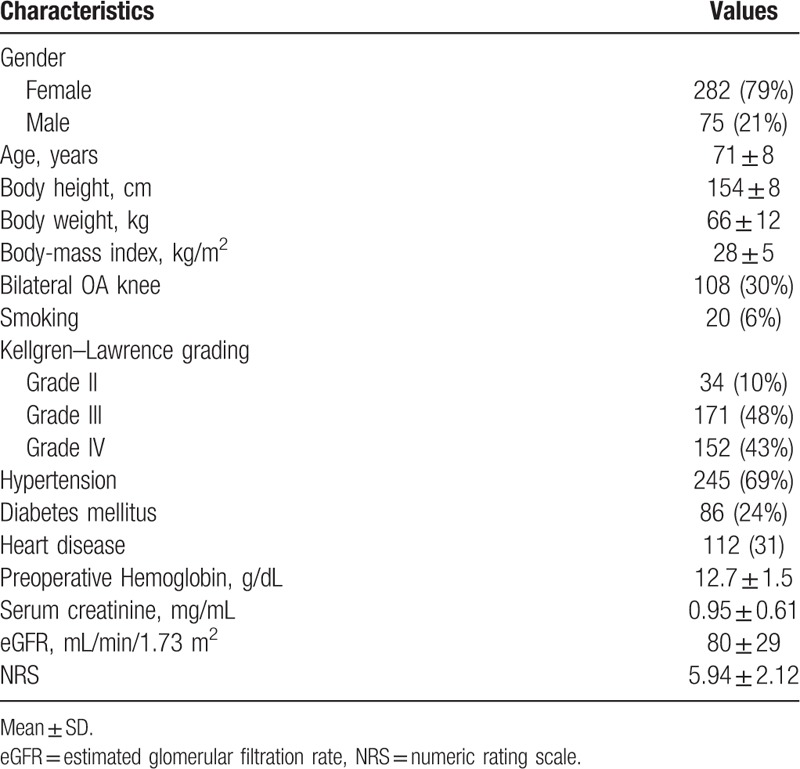
Baseline characteristics of knee osteoarthritis (OA) patients.

### 3.2 Patients with OA who required to receive TKR tended to have moderate or severe knee pain

**Figure 1:**
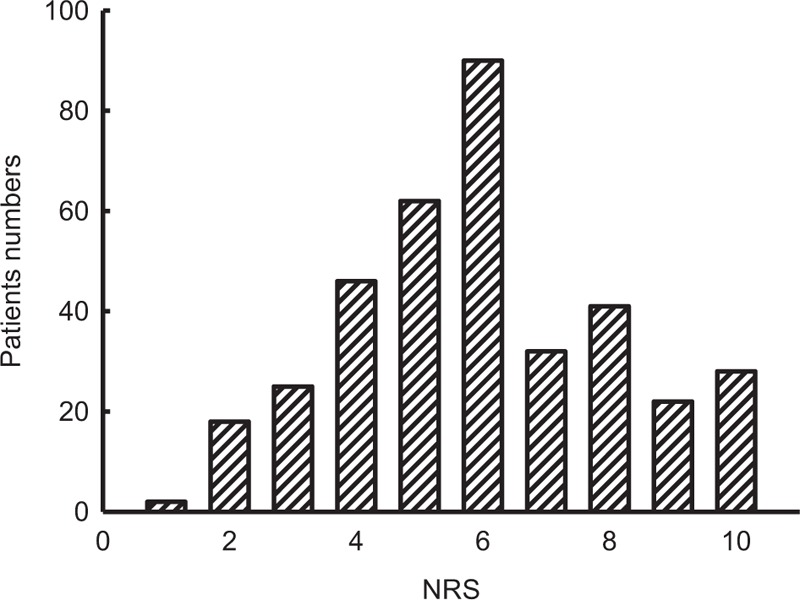
Distribution of knee pain scores according to a 10-point numeric rating scale (NRS) as self-defined by knee osteoarthritis patients.

Figure 1 shows the distribution of knee pain scores in all subjects. Based on NRS scores, they were further divided into 3 groups by knee pain severity: mild, moderate, and severe pain. In this study, more than 87% of subjects suffered from moderate to severe knee pain. The percentages of mild, moderate, and severe knee pain were 12.3%, 54.3%, and 33.4% respectively. This result showed that subjects with knee OA and required to receive TKR, tended to have at least moderate knee pain.

### 3.3 Patients with severe knee pain tended to have renal insufficiency

Table 2 revealed the demographic data and clinical characteristics in the 3 groups categorized by pain severity. There were no significant differences in age, gender, bilateral OA knee involvement, distribution of K/L grades of knee OA, or medical coexisting diseases, within each knee pain group. However, compared to the mild and moderate knee pain groups, patients with severe knee pain had a lower BMI value (27.2 ± 3.8, *P* < .01), higher serum creatinine level (1.1 ± 0.9, *P* < .01) and lower eGFR (73.3 ± 29.0, *P* < .05).

**Table 2:**
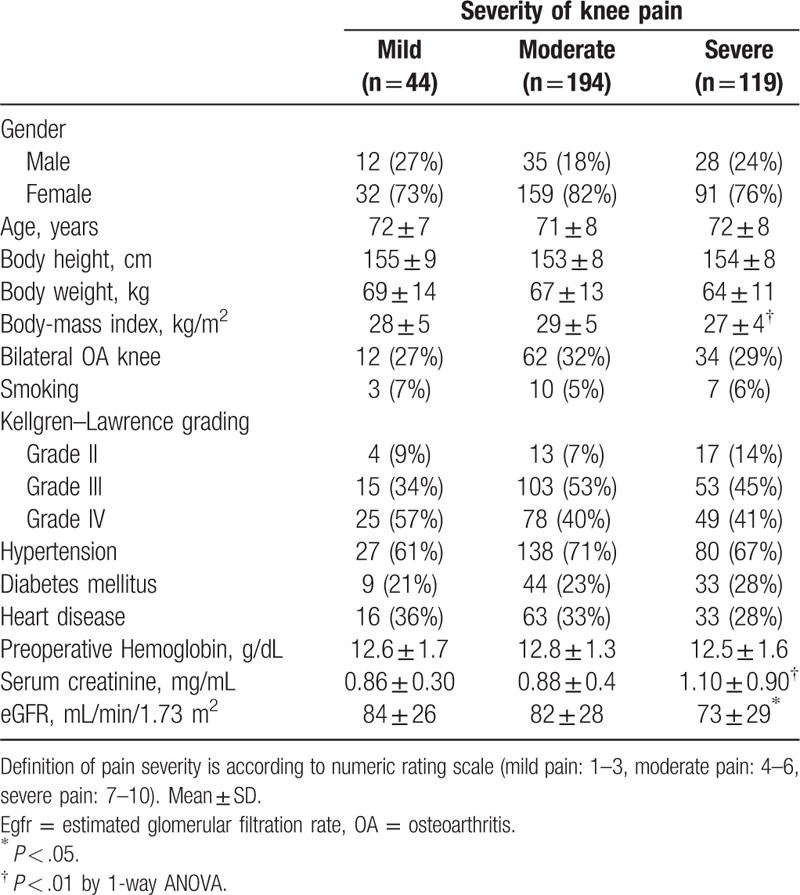
Baseline characteristics of osteoarthritis (OA) knee patients by severity of knee pain.

**Figure 2:**
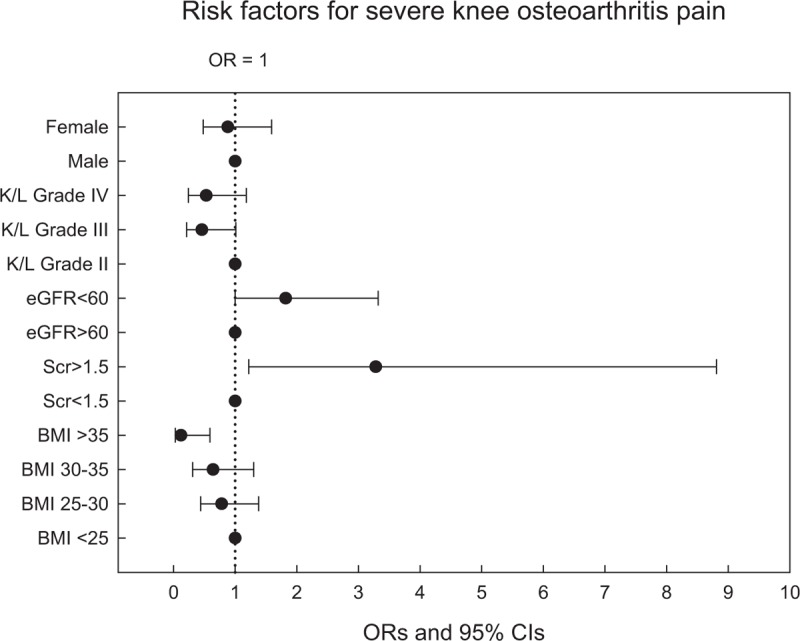
Odds ratios of association factors for severe knee osteoarthritis pain. Odds ratios derived from multivariate binary logistic regression analysis, depicting the strength of association between severe OA pain and association factors (included female, higher K/L grade, renal insufficiency, and higher BMI). The point estimates and 95% confidence intervals are shown, and a larger odds ratio indicates a stronger association between the association factors and severe knee OA pain. BMI = body-mass index, OA = osteoarthritis.

The multivariate binary logistic regression for evaluating association factors associated with knee pain also revealed similar results. As shown in Figure 2 and Table 3, renal insufficiency defined as serum creatinine >1.5 mg/dL and an eGFR of <60 mL/min per 1.73 m^2^ had higher ORs for severe knee pain (OR = 3.28, 95% confidence interval [CI] 1.22–8.81, *P* < .05; OR = 1.82, 95% CI 1.00–3.32, *P* < .05, respectively). While morbid obesity (BMI >35 kg/m^2^) was likely to have a lower risk for developing severe knee pain (OR = 0.12, 95% CI 0.03–0.59, *P* < .01).

**Table 3:**
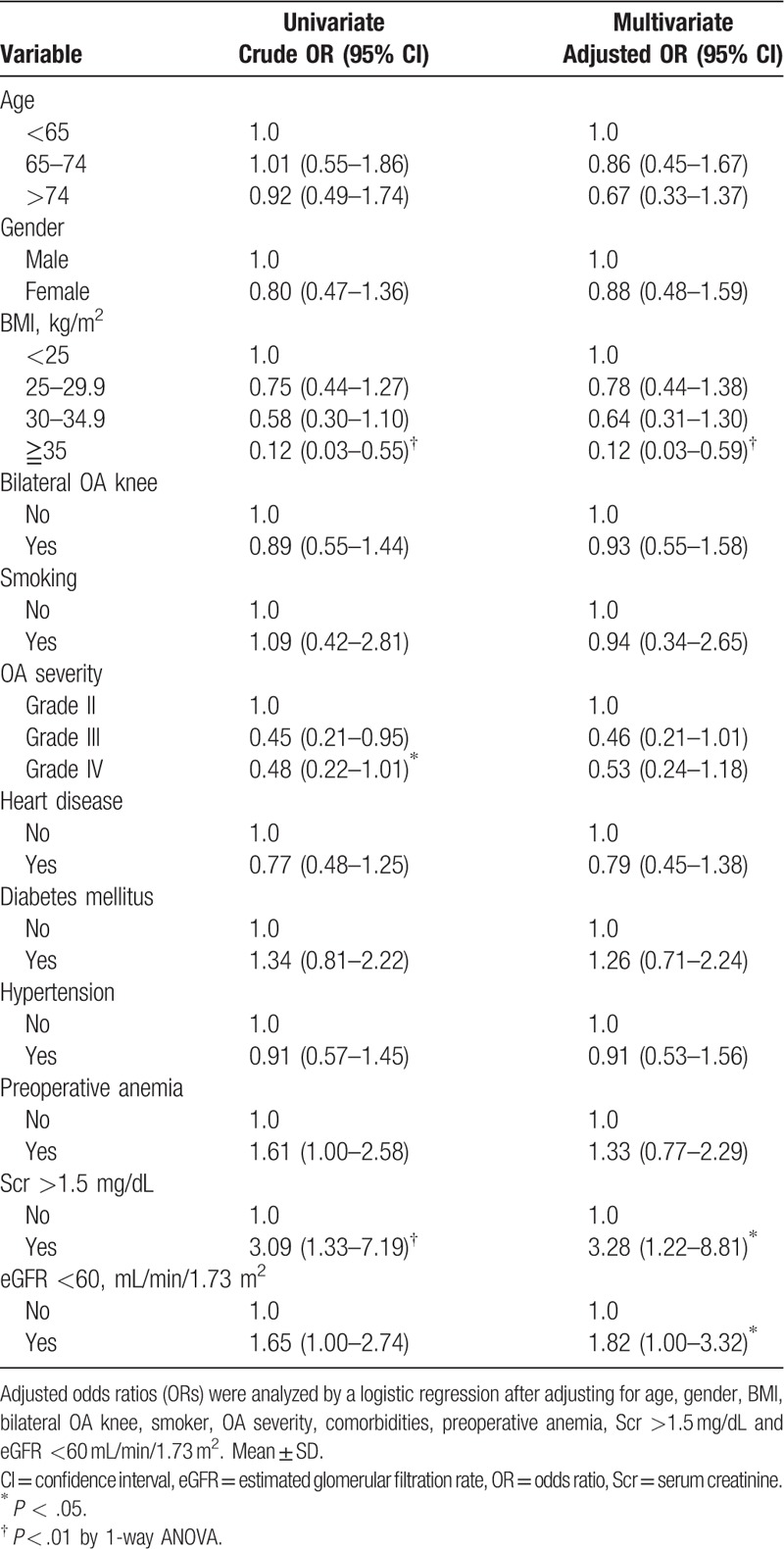
Association factors for severe knee osteoarthritis (OA) pain.

These results were further approved by Pearson correlation test. Table 4 shows weak but significantly correlations of serum creatinine, and the eGFR with pain score (Pearson correlation coefficient *r* = 0.13, *P* = .02 and *r* = −0.11, *P* = .04, respectively). However, other continuous variables showed no significant correlation with the knee pain score.

**Table 4:**
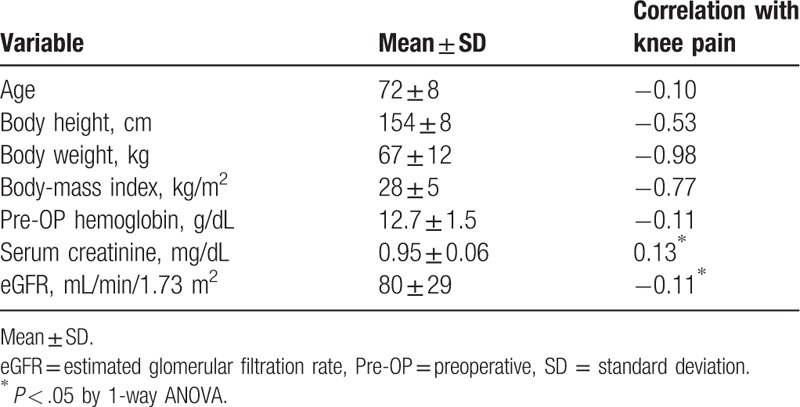
Pearson correlations between knee pain and association factors.

### 3.4 Higher K/L grades were associated with higher BMI

Patient's demographics data and clinical characteristics by 3 K/L grading groups are described in Table 5. Although the severity of knee OA radiographic grading did not correlate with the severity of pain, a significant association with BMI was noted. Comparing with K/L grade II, patients with more severe radiographic OA had higher BMI. The BMI in K/L grade II was 26.1 ± 3.8, while K/L grade III and IV were 28.3 ± 4.3 and 28.5 ± 4.7, respectively, *P* < .05. None of the remaining variables show a significant difference among the 3 groups.

**Table 5:**
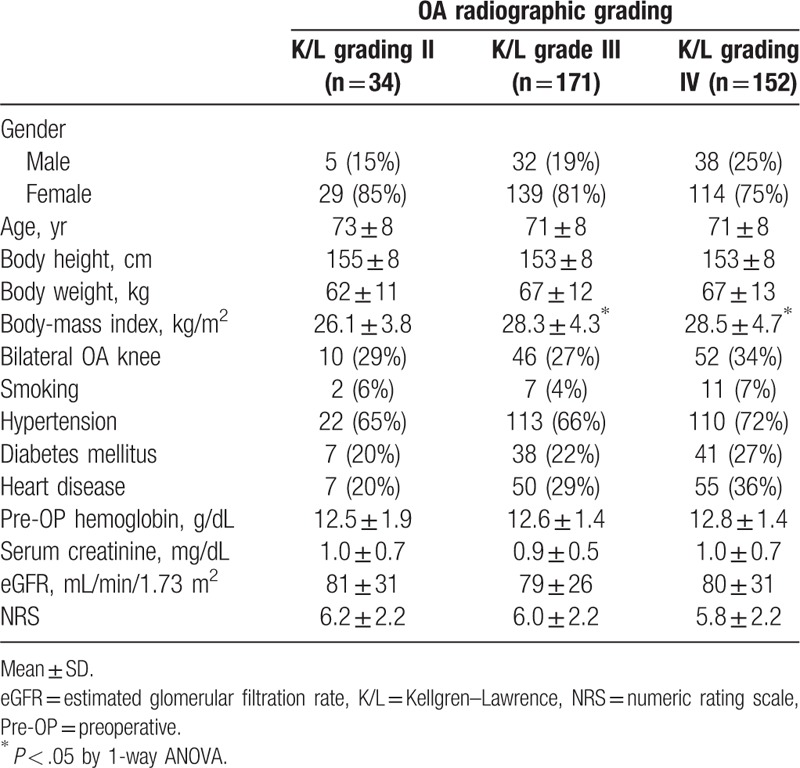
Baseline characteristics of osteoarthritis (OA) knee patients by OA radiographic grading.

## 4 Discussion and conclusions

Our present study examined factors associated with knee OA pain. We analyzed data from patients with end-stage knee OA who received TKR. Data of the National Health Insurance Research Database (NHIRD) in Taiwan from 2000 to 2005 showed that 35,805 patients received TKR surgery, at an average age of 72 years, and that 25,963 (72.51%) were female patients. Comparing our study findings with data from the NHIRD, we selected 357 OA knee patients from 1 medical center, at an average age of 71.47 years, and 282 (79%) patients were female. Therefore, we contend that our study patients can represent general Taiwanese knee OA patients. Patients with total knee arthroplasty are to alleviate OA-induced pain, to restore knee function, or to improve life quality. The NRS is an index scoring the patients’ knee pain severity. Pain is one of critical causes driving patients to undergo total knee arthroplasty. In this study, there are 44 patients with mild severity of knee pain but operating total knee arthroplasty because of other causes because they would like to repair knee function or expand quality of life.

In this study, we found that elevated serum creatinine and a lower eGFR were significantly associated with severe knee OA pain (*P* < .05). Significant correlations of pain score with serum creatinine and eGFR were also approved by Pearson correlation test. According to the clinical guidelines of the American Academy of Orthopedic Surgeons, NSAIDs are recommended as the initial drug to treat persistent knee OA pain.^[22]^ Currently, selective cyclooxygenase (COX)-2 inhibitors are commonly used as an anti-analgesic drug for pain management because it avoids upper gastrointestinal bleeding complications, but a nephrotoxic effect is still a concern.^[23,24]^ In a previous study, Koseki et al reported that 14 of 235 early rheumatic arthritis patients on NSAIDs suffered from mild reversible elevated serum creatinine.^[25]^ It is well established that older adults are at higher risk of NSAID-induced renal insufficiency.^[26]^ Due to the renal toxicity of NSAIDs, patients who have been prescribed with a higher dose of NSAIDs for severe knee pain might develop renal insufficiency after a long-term exposure to high dose NSAIDs.^[27]^ In this study, most knee OA patients received COX-2 inhibitors for pain relief before receiving TKR. The positive correlation between serum creatinine and pain scores and the negative correlation between the eGFR and pain scores might be possibly associated with the use of NSAIDs. Although currently, most patients have subclinical renal insufficiency, chronic renal failure might be developed under long-term analgesic medication for pain relief. Another possible explanation is a lower dosage or even avoidance of analgesics might be prescribed in knee OA patients with renal insufficiency. Thus, inadequate pain relief might be encountered in those patients and led to more severe pain. Furthermore, glucosamine, a common symptomatic treatment for OA can also cause apoptosis in renal tubular and mesangial cells in experimental animal studies.^[28,29]^ The introduction of glucosamine resulted in loss of renal function has also been reported previously.^[29,30]^ This renal toxicity of glucosamine might also involve in the development of renal insufficiency in patients with severe OA pain. It need further studies to confirm why renal insufficiency was associated with severe knee pain. We should care more about their medication application for those patients with greater pain due to OA of the knee.

We found a significant difference between OA radiographic K/L grading groups in terms of the BMI. A higher BMI was associated with a greater K/L grading score as shown in Table 5. Obesity is considered a risk factor for knee OA.^[31,32]^ The knee is a weight-bearing joint, and excess body weight persistently causes physical overloading of joints, hence can cause knee cartilage fragmentation and development of knee OA. Our present study shows that higher K/L grades were associated with higher BMI. In contrast, the K/L imaging grades were not correlated with the NRS values. A higher BMI is a well-known factor related to symptomatic and radiographic OA knee.^[33]^ Our study had similar results as the previous literature. In this study, 34 patients with grade II OA underwent total knee arthroplasty. Among them, 88% of the OA patients expected to reduce knee pain after total knee arthroplasty. The other patients underwent total knee arthroplasty because they anticipated to restore knee function or to improve quality of life.

Our present results also demonstrate a significant difference between knee pain severity in terms of BMI. Inversely, severe knee pain was associated with a lower BMI, compared to other 2 groups. In addition, our results show that morbid obesity (BMI ≧35 kg/m^2^) was found to have a significantly reduced risk of developing severe pain (OR = 0.12, *P* < .01). However, no significant correlation was found between the BMI and knee pain severity by Pearson correlation. Previous studies showed that obesity is a strong predictor of knee pain. Jhun et al pointed out that obesity is a factor for knee pain and pain severity.^[17]^ Creamer et al also disclosed that the BMI is an important factor in determining the severity of knee OA pain.^[34]^ Disagreement in our results between knee pain severity and the BMI may have been due to limited daily functions in knee OA patients with a higher BMI and greater knee joint damage. In our retrospective study, we could not further distinguish the pain scores reported were at rest or during movement. Since Knee pain is commonly induced by specific activities such as climbing stairs and walking, and these activities would be difficult for extremely obesity patients. Thus, less knee pain reported by those patients with higher BMI might be related to less daily activities.

As shown in Figure 2 and Table 5, no association between radiographic OA images and severe pain severity was found in our study. Knee pain may arise from synovitis or bone marrow edema that cannot be interpreted on plain x-ray films.^[35,36]^ Advanced imaging studies such as magnetic resonance imaging and bone scintigraphy, were shown to be correlated to knee OA pain severity.^[37,38]^ An extra-articular structure as the knee pain source was also reported in a recent study.^[39]^ In clinical practice, knee OA patients with the same K/L grading may complain of different knee pain severities. A female gender was reported to be a well-known risk factor of knee OA.^[40,41]^ It was compatible with our results. In this study, the majority of patients who received TKR were females. However, female was not a risk factor of severe pain. Although a higher proportion of females was demonstrated in each knee pain group, we demonstrated that females were not significantly associated with severe knee OA pain, compared to mild to moderate knee OA pain. The study findings might be explained by females having more knee pain, but not specific to any knee severity.

### 4.1 Limitations of this study

Readers are warned against over-interpreting our study findings because this study had 3 major limitations. In this retrospective study, knee OA patients defined the worst knee pain themselves by the NRS. But knee pain is not defined as to how it was induced such as at rest, walking, or climbing stairs. And almost all subjects were prescribed anti-analgesic drugs until the day before the operation, so we could not rule out the effect of drugs on pain severity. Using only a single pain scale to define knee pain severity by patients might not be totally precise, and another pain scale such as Western Ontario and McMaster Universities Osteoarthritis Index could simultaneously be used to define the pain scale in future studies.

## Acknowledgments

The authors express their gratitude to Dr. Yen-Kuang Lin, a statistical expert of Research Center of Biostatistics, College of Management, Taipei Medical University, Taipei, Taiwan, for assistance of data analyses.

## Author contributions

**Conceptualization:** Chung-Ching Chio, Man-Kit Siu, Yu-Ting Tai, Tyng-Guey Chen, Wei-Pin Ho, Ruei-Ming Chen.

**Data curation:** Chung-Ching Chio, Man-Kit Siu, Yu-Ting Tai, Tyng-Guey Chen, Wei-Pin Ho, Jui-Tai Chen, Hsiao-Chien Tsai, Ruei-Ming Chen.

**Formal analysis:** Man-Kit Siu, Yu-Ting Tai, Jui-Tai Chen, Hsiao-Chien Tsai, Ruei-Ming Chen.

**Funding acquisition:** Chung-Ching Chio, Ruei-Ming Chen.

**Investigation:** Chung-Ching Chio, Man-Kit Siu, Yu-Ting Tai, Tyng-Guey Chen, Wei-Pin Ho, Ruei-Ming Chen.

**Methodology:** Man-Kit Siu, Yu-Ting Tai, Wei-Pin Ho, Jui-Tai Chen, Hsiao-Chien Tsai, Ruei-Ming Chen.

**Project administration:** Ruei-Ming Chen.

**Resources:** Chung-Ching Chio, Yu-Ting Tai, Tyng-Guey Chen, Ruei-Ming Chen.

**Supervision:** Chung-Ching Chio, Tyng-Guey Chen, Wei-Pin Ho, Ruei-Ming Chen.

**Validation:** Chung-Ching Chio, Man-Kit Siu, Jui-Tai Chen, Ruei-Ming Chen.

**Visualization:** Yu-Ting Tai, Hsiao-Chien Tsai, Ruei-Ming Chen.

**Writing – original draft:** Chung-Ching Chio, Man-Kit Siu, Yu-Ting Tai, Ruei-Ming Chen.

**Writing – review & editing:** Chung-Ching Chio, Man-Kit Siu, Yu-Ting Tai, Tyng-Guey Chen, Wei-Pin Ho, Jui-Tai Chen, Hsiao-Chien Tsai, Ruei-Ming Chen.
